# Differential regulation of Treg stability in human naïve and effector Treg subsets by TGFβ-signaling via ARKADIA-SKI axis

**DOI:** 10.3389/fimmu.2025.1636434

**Published:** 2025-09-09

**Authors:** Fan Yang, Alexis Yanes, Miao Li, Patrick Heizer, Ivan Linatoc, Michael E. Stephens, Yang Song, Tatiana Ort, Kyle J. Bednar, Yasuhiro Ikeda, Zengli Guo

**Affiliations:** ^1^ Biologics Engineering, Oncology R&D, AstraZeneca, Gaithersburg, MD, United States; ^2^ Bioscience Immunology, Research and Early Development, Respiratory and Immunology, Biopharmaceuticals R&D, AstraZeneca, Gaithersburg, MD, United States

**Keywords:** Treg, TGFβ, autoimmune, naïve/effector Treg cell, Ski, Arkadia

## Abstract

**Introduction:**

The human FOXP3^+^ regulatory T (Treg) cells, a subset of CD4^+^ T cells with immunosuppressive function, are essential for the maintenance of immune homeostasis and tolerance. Treg cells are a heterogeneous population, subdivided into a less stable “effector” subset and a more stable “naïve” subset. Under inflammatory conditions, Treg cells can lose their immunosuppressive properties, contributing to the development of autoimmune diseases. The TGFβ signaling is pivotal for the induction of Treg cells from naïve CD4 T cells and the thymic development of natural Treg cells. However, how TGFβ signaling regulates established naïve and effector Treg cells is not fully understood.

**Methods:**

Human naïve and effector Treg cells were isolated from healthy donors using flow cytometry. Different subsets of Treg cells were treated with a TGFβ inhibitor or genetically modified to express SKI or ARKADIA via lentiviral transduction. Treg cell phenotype, stability and signaling regulation were analyzed using flow cytometry, western blotting, transcriptomic and meta-analyses. The functionality of Treg cells was analyzed by *in vitro* coculture assays.

**Results:**

We find that the TGFβ signaling is differentially regulated in Treg subsets, with higher activity in the naïve Treg subset. Blockade of TGFβ pathway destabilizes both naïve and effector Treg cells, disrupting their immunosuppressive functions, with effector Treg cells being more susceptible. Further analysis shows that naïve Treg cells express lower levels of SKI protein, a negative regulator of TGFβ signaling suppressed by TGFβ-induced protein degradation. SKI overexpression destabilizes Treg cells and disrupts their immune suppressive function. Transcriptomic and meta-analyses reveal that TGFβ blockade and SKI overexpression commonly modulate pathways crucial for Treg to effector T cell conversion, downregulating Treg signature genes and upregulating effector T cell markers, which are validated as potential SKI targets. Importantly, overexpression of ARKADIA, an E3 ubiquitin ligase of SKI, efficiently reduces SKI levels, enhancing Treg cell stability and functionality under both TGFβ inhibition and chronic proinflammatory cytokine stimulation.

**Conclusion:**

Our results identify a previously unrecognized role of the TGFβ-ARKADIA-SKI axis in regulating the stability and functionality of human Treg subsets, highlighting novel strategies for harnessing TGFβ-associated pathways to stabilize human Treg cells for clinical applications.

## 1 Introduction

The CD4^+^FOXP3^+^ regulatory T (Treg) cells play a vital role in maintaining immune homeostasis and tolerance. Treg cells comprise only ~10% of CD4^+^ T cells, they are a heterogenous population and can be further subdivided into a less stable “effector” subset and a more stable “naïve” subset (1). Naïve Treg cells, constituting only 0.2 to 3.3% of the total circulating CD4^+^ T cells, are thymus-derived and have not experienced antigen stimulation. In contrast, effector Treg cells are antigen-experienced and can originate either from the thymus or through convention from CD4^+^ T cells. Naïve Treg cells can convert to effector Treg cells upon TCR stimulation (2, 3). Compared with effector Treg cells, naïve Treg cells exhibit less proliferation but greater stability in suppressive function and FOXP3 expression (4–6). One of the unique characteristics of Treg cells is their plasticity, which is defined as the ability to adapt to distinct inflammatory cues while retaining their suppressive capacity (7). Reports show that FOXP3^+^ Treg cells can co-express signature transcription factors of other T helper subsets, such as T-bet for Th1 cells, GATA-3 for Th2 cells, Bcl6 for T_FH_ cells and RORγt for Th17 cells. The co-expression of these transcription factors in Treg cells confers their advantage of lineage specific suppression, but also poses risks of Treg instability, which leads to the loss of immunosuppressive function and potentially contributing to autoimmunity (8–10). The study of regulatory mechanism of Treg plasticity and stability will provide valuable insights on the onset and pathogenesis of autoimmune diseases.

The immune suppression of Treg cells is achieved by generating an inhibitory environment, which involves the secretion of inhibitory cytokines (such as IL10, IL35 and TGFβ), metabolites (such as adenosine), cytotoxic granzymes and perforin, in addition to consuming local IL2 (11). Treg cells can directly inhibit T cells or antigen presenting cells (APCs), through the expression of negative regulatory cell surface receptors such as cytotoxic T lymphocyte antigen 4 (CTLA-4) (11). With these unique features, adoptive Treg cell therapy has recently emerged as an innovative approach to treat severe autoimmune diseases and other immunological diseases, with its most recent pre-clinical and clinical practice summarized by Bluestone et al. (12). This therapy involves the isolation of Treg cells, expanding them *ex vivo*, engineering with Chimeric Antigen Receptor (CAR) or antigen specific TCR to enhance therapeutic specificity and potency, and eventually reinfusing them to restore immune tolerance (12). One major challenge for Treg cell therapy is the concern of their stability, which is defined by the maintenance of three critical traits: (a) stable FOXP3 expression, (b) efficient suppressive activity, and (c) absence of effector activity (13). An increasing number of clinical trials are opting to use naïve Treg cells as a modality due to their greater stability compared to the total bulk Treg population, which was commonly used in previous studies. (6, 12). However, the underlying differences and mechanisms of regulating naïve Treg stability are largely unclear. Moreover, there is growing interest in exploring whether the stability of naïve Treg cells can be further enhanced via genetic engineering.

Transforming growth factor β (TGFβ) plays important roles in Treg biology, including induced Treg differentiation, thymic Treg development and immune suppressive function (14). TGFβ is secreted as a latent form, complexed with a latency-associated peptide (LAP), and is activated by the cleavage of LAP through various mechanisms, including integrins and proteases (15). TGFβ binds its receptor, TGFβRII which then recruits TGFβRI. This triggers phosphorylation events that activate receptor-regulated SMADs (R-SMADs), including SMAD2 and SMAD3. These SMADs translocate to the nucleus and work together with the common-partner SMAD4 (co-SMAD) to modulate downstream gene expression (15). The TGFβ signaling is negatively regulated by SKI proto-oncogene (SKI), its homolog SKI like proto-oncogene (SKIL/SNON) and Inhibitory SMADs (I-SMADs, including SAMD6 and 7). These elements act as negative regulatory factors by recruiting transcriptional corepressors, such as histone deacetylases (HDACs) (16, 17). The SKI and SNON protein can also be modulated by TGFβ signaling via E3 ubiquitin ligase AKADIA mediated protein degradation (18–20). TGFβ signaling is critical for the induction of FOXP3 expressing Treg cells from naïve CD4 T cells (21, 22) and thymic Treg development (23, 24). However, the role of TGFβ in the maintenance of established Treg cells is less clear, with studies primarily conducted in mouse models indicating contentious and ambiguous findings (25–28). The study of TGFβ signaling in human Treg cells is limited due to the heterogeneity of human Treg population and technical challenges in the isolation of Treg subsets. With rapid progress of the Treg cell therapies in clinical development, it is particularly beneficial to investigate how TGFβ signaling regulates the stability and functionality of human Treg cells, especially different subpopulation.

In this study, we report a previously unappreciated role of TGFβ signaling in human naïve and effector Treg populations and demonstrate that TGFβ maintains the identity of human naturally occurring Treg cells through the ARKADIA-SKI axis, providing evidence that suggests novel therapeutic strategies for enhancing Treg stability.

## 2 Materials and methods

### 2.1 Human Treg cell isolation, culture and TGFβR1 inhibitor treatment

Leukopaks from healthy donors were obtained from StemExpress, BioIVT, or AllCells, with their full consent, under the approval of Institutional Review Board obtained by these companies. PBMCs were treated with ACK lysis to remove residual red blood cells, followed by positive selection for CD25^+^ cells (Miltenyi Biotec, 130092983). These enriched CD25^+^ cells were sorted by FASC to isolate CD4^+^CD25^+^CD127^−^CD45RO^-^ naïve Treg cells or CD4^+^CD25^+^CD127^−^CD45RO^+^ effector Treg cells on FACSAria™ II or FACSAria™ Fusion (BD Biosciences) with the indicated antibodies. The isolated primary cells were resuspended at 0.5 x 10^6/ml in RPMI1640 (Gibco, A1049101), supplemented with 10% HI-FBS (Gibco, 10500064), 1x Penicillin-Streptomycin-Glutamine (Gibco, 10378016), 1x 2-Mercaptoethanol (Gibco, 21985023), 1x Gibco^®^ MEM Non-Essential Amino Acids (Gibco, 11140050), 1000 IU/ml recombinant hIL-2 (Peprotech, 200-02-1MG), and 2nM rapamycin (Sigma, R8781). Activation was done using Dynabeads™ Human Treg Expander (Thermo Fisher, 11129D) in a cell-to-bead ratio of 1:4. Every 2–3 days, cells were passaged and media refreshed, with expansion continuing for a total of 14 days. Post-expansion, cells were collected by removing Dynabeads, then either cryopreserved or directly subjected to treatments. For transductions and most treatments, expanded Treg cells, whether ongoing or retrieved from cryo-stock, were maintained at 0.5 x 10^6 cells/ml in the same medium without rapamycin, with passaging every 2–3 days. Activation was done at a 1:1 cell-to-bead ratio every 14 days. TGFβR1 inhibitor treatments were done in the presence of 1nM TGFβR1 inhibitor SB431542 (Selleckchem, S1067), which was replaced every 3 days.

### 2.2 Flow cytometry

Fluorescence-conjugated antibodies for CD25 (BC96, 302606), CD127 (A019D5, 351310), CD45RO (UCHL1, 304234), FOXP3 (206D, 320114), HELIOS (22F6, 137214), IL17A (BL168, 512338), IFNγ (4S.B3, 502532), were purchased from Biolegend, and CD4 (L200, 552838), CD45RA (HI100, 568557) were from BD Biosciences. For FACS analysis, 0.5–1 × 10^5 cells were collected and subjected to surface staining, followed by intracellular staining using the eBioscience™ Foxp3/Transcription Factor Staining Buffer Set (Thermofisher, 00-5523-00) according to the manufacturer’s instructions. For intracellular cytokine staining, cells were stimulated for 4 hours with 200 ng/ml PMA (Sigma, 19-144), 5 μg/ml BFA (Sigma, B7651), and 1 μM Ionomycin (Sigma, I0634). Stained cells were analyzed using BD^®^ LSR II, LSRFortessa™, FACSymphony™ A3 or FACSymphony™ A5 instruments (BD Biosciences). The FACS data were analyzed using FlowJo software (TreeStar).

### 2.3 Plasmids, lentivirus transduction and MACS enrichment

The coding sequence (CDS) of human SKI (NM_003036.4), or mutant (P35S) SKI (c.[97GGCGGCCCG>GGAGGATCC] on NM_003036.4), or ARKADIA/RNF111 (NM_001270528.2) was inserted upstream of the truncated NGFR sequence (29), flanked by T2A in a lentiviral vector driven by EF1 promoter. Subclones were generated by Genscript. Lentivirus was produced using the pPACKH1 packaging system (System Biosciences, cat# LV500A-1) according to the manufacturer’s instructions. For transduction, 14-day expanded Treg cells, whether ongoing or retrieved from cryo-stock, were activated with Dynabeads™ Human Treg Expander (Thermo Fisher, 11129D) at a cell-to-bead ratio of 1:1 for one day. This was followed by a 2-hour spin inoculation of lentivirus in the presence of 10 μg/mL protamine sulfate. The infected cells were incubated overnight with the virus in a 37 °C CO2 incubator before replacing with fresh media. 7 days after infection, the transduced cells were enriched for NGFR^+^ populations, using MACSelect™ LNGFR MicroBeads (Miltenyi Biotec, 130091330) as per the manufacturer’s instructions. The enriched NGFR+ cells were assessed for enrichment efficiency by flow cytometry, activated and subjected to subsequent treatments for the desired duration.

### 2.4 Treg de-stabilization assay

Treg cells were cultured in media with or without the following cytokines: 20 ng/ml IL-1β (Peprotech, 200-01B-10UG), 20 ng/ml IL-6 (R&D, 206-IL-010), and 40 ng/ml IL-23 (Biolegend, 574104). The culture duration spanned 3 to 5 weeks, with media being refreshed every 2–3 days. Treg identity was assessed weekly using flow cytometry.

### 2.5 *In vitro* Treg suppression assay

Naïve CD25^-^CD4^+^ effector (Teff) cells were derived from the CD25^-^ PBMC remaining after Treg isolation, using negative selection to enrich CD4^+^ cells (Miltenyi Biotec, 130045101). These cells were cryo-reserved until usage. For evaluating Treg *in vitro* suppression ability, Teff cells were retrieved from cryo-stock and labeled with CellTrace™ Violet (CTV) (Invitrogen, C34557) as per the manufacturer’s instructions. A fixed amount of 5 × 10^4^ labeled Teff were mixed with varying amounts of Treg cells at different ratios, in Treg media excluding IL2, with the presence of human Treg Suppression Inspector (Miltenyi Biotec, 130092909) at a Teff-to-bead ratio of 1:1. The cell mixtures were cultured for 4 days at 37 °C CO2 incubator. Following incubation, the proliferation of Teff cells was assessed by measuring CTV dilution via flow cytometry, analyzed using FlowJo software. Suppression efficiency was calculated as “% inhibition of Teff division,” determined by the percentage reduction in the division index of Teff cells compared to Teff cells cultured alone.

### 2.6 Western blot

Treg cell pellets were lysed in RIPA buffer (Thermo Scientific, 89900) containing 1x Halt™ Protease Inhibitor Cocktail (Thermo Scientific, 78430), in 4°C with rotation for 20min. The lysates were then centrifuged at 14,000 g for 10 minutes, and the supernatant was collected and were boiled in 1x Laemmli Sample Buffer sample buffer (Bio-rad, 1610747) with 1x NuPAGE™ Sample Reducing Agent (Invitrogen, NP0004) at 70°C for 10min. The prepared samples were subjected to electrophoresis using the Invitrogen™ NuPAGE™ gels and XCell SureLock Mini-Cell system (Invitrogen), followed by transfer using the iBlot™ 2 Transfer system (Invitrogen). The samples were then immunoblotted with the following primary and secondary antibodies: SKI (Santa Cruz, G8, sc-33693), RNF111 (Abnova, 1C4, H00054778-M05), GAPDH (Cell Signaling Technology, 5174), Anti-mouse IgG HRP-linked (Cell Signaling Technology, 7076S), and Anti-rabbit IgG HRP-linked (Cell Signaling Technology, 7074S). Protein levels were quantified using ImageJ. For the original raw gel images of western blot shown in figures, see **Supplementary Material**.

### 2.7 Bulk RNA-seq sample preparation and analysis

For TGFβi treatment in freshly isolated naïve and effector Treg cells, cells from FACS were directly cultured in the absence of rapamycin, with or without 1nM SB431542 (Selleckchem, S1067) for 5 weeks before RNA extraction. For (P35S) SKI overexpression in naïve Treg cells, expanded naïve Treg cells were transduced, NGFR^+^ cells enriched, and cultured for 7 days prior to RNA extraction.

Total RNA was extracted using the MagBind Total RNA 96 kit (Omega Biotek) according to the manufacturer’s instructions. RNA quality and quantity were assessed with the Qubit HS RNA assay (ThermoFisher) and the Tapestation High Sensitivity RNA assay (Agilent). From each sample, 100 ng of total RNA was used for library preparation. Briefly, human ribosomal RNA was depleted using the Ribo-Zero Plus rRNA Depletion Kit (Illumina), followed by library generation with the NEBNext Ultra II RNA Directional Kit (New England Biolabs), according to the manufacturer’s instructions. Sequencing was performed on an Illumina NovaSeq 6000 sequencer with 2×150 bp paired-end reads. Following quality control and adapter trimming of the raw sequencing data, the reads were then aligned to the human reference genome (GRCh38) using STAR (version 2.7.11b) under default settings. Gene-level read counts were extracted from STAR’s ‘ReadsPerGene’ output files, and the gene count matrix was prepared for downstream analysis.

Differential gene expression analysis was performed using the DESeq2 package (30) in R. The raw read count matrix obtained from STAR was imported into DESeq2 and subjected to normalization using the DESeq2 normalization method to account for differences in sequencing depth across samples. To assess the variance in gene expression profiles among samples, a Principal Component Analysis (PCA) was performed. Specifically, the DESeqDataSet object containing the raw read counts was transformed using the variance stabilizing transformation (VST) function to stabilize variance across the expression data. PCA was conducted on the transformed dataset, and the PCA plot was generated using the plotPCA function, which computes and plots the first two principal components, representing the greatest variance within the dataset. Paired comparisons between experimental groups were conducted to identify differentially expressed genes. A threshold of a fold change of 2 was used to define biologically significant changes in gene expression. Gene Set Enrichment Analysis (GSEA) was performed using the clusterProfiler package (31) in R to identify enriched biological pathways in differentially expressed genes. A ranked list of genes based on log2 fold changes from DESeq2 was used as input for the analysis. Statistical significance was assessed using the Wald test, and p-values were adjusted for multiple comparisons using the Benjamini-Hochberg method to control the false discovery rate (FDR). Genes with an adjusted p-value of less than 0.05 were considered significantly differentially expressed. For the raw gene expression profiles, see **Supplementary Material**.

### 2.8 Statistical analysis and bioinformatics

Unless otherwise indicated, statistical significance was determined by Student t test for two-sample analyses, analyzed using GraphPad Prism. P < 0.05 was considered as statistically significant and labeled with one asterisk.

GSEA and PCA analyses were done using R package “clusterProfiler”. Heatmaps were generated using R package “pheatmap” or a web tool Morpheus (https://software.broadinstitute.org/morpheus). Venn diagrams were plotted using R package “VennDiagram”. The SKI CHIP-seq data was retrieved from GSE107556 and visualized using IGV browser (32).

## 3 Results

### 3.1 Human Treg subsets demonstrate differential TGFβ signaling and stability

**Figure 1:**
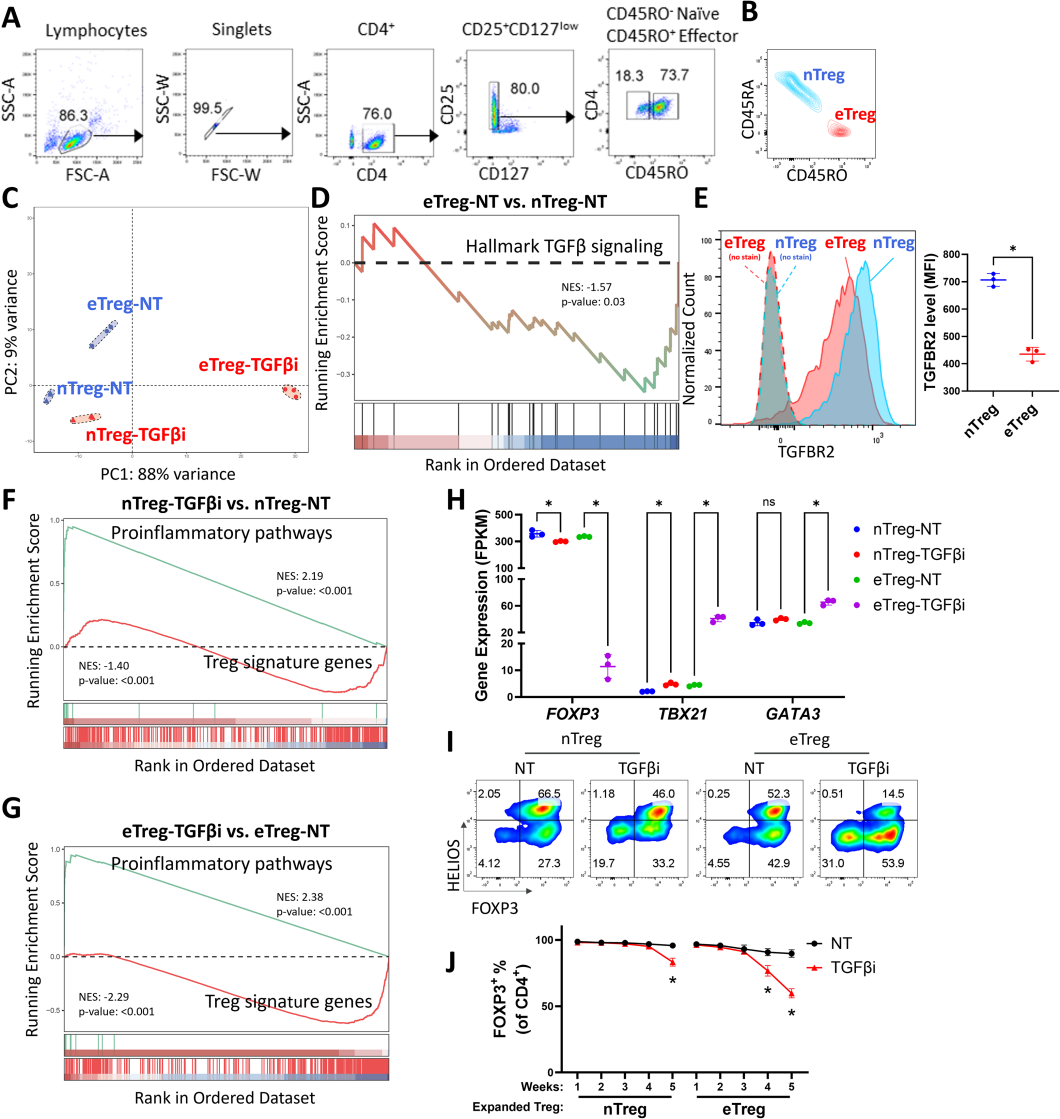
TGFβ signaling blockade destabilizes human Treg cells. **(A)** A diagram of the gating strategy to isolate naïve Treg (nTreg, CD4^+^CD25^+^CD127^−^CD45RO^-^) cells and effector Treg (eTreg, CD4^+^CD25^+^CD127^−^CD45RO^+^) cells from CD25 beads enriched PMBC in healthy donors by FACS. The flow cytometry profiles are representative of more than three donors. **(B)** The expression of CD45RO and CD45RA in freshly isolated nTreg and eTreg cells were assessed by flow-cytometry. The flow cytometry profiles are representative of more than three donors. **(C)** Principal Component Analysis (PCA) of whole genome transcriptome data in nTreg and eTreg cells with (TGFβi) or without (NT) TGFβR1 inhibitor treatment. n = 3 replicates of one donor. **(D, F, G)** Gene set enrichment analysis (GSEA) was performed on the indicated sample groups and gene collections. Normalized Enrichment Scores (NES), and p-values were calculated using permutation tests and are labeled accordingly. **(E)** The expression of TGFBR2 in freshly isolated nTreg and eTreg cells were assessed by flow-cytometry (Left panel). The flow cytometry profiles are representative of three donors. Quantifications of MFI of TGFBR2 level in nTreg and eTreg cells (Right panel). Data are presented as Mean ± SD. ^∗^p < 0.05, two-sided t test. n = 3 replicates of one donor. This data is representative of experiments conducted in three donors. **(H)** A scatter plot illustrates gene expression levels of the indicated genes from whole genome transcriptome dataset. Data are presented as Mean ± SD. ^∗^p < 0.05, two-sided t test. n = 3 replicates of one donor. **(I)** Representative flow cytometry profiles showing FOXP3 and HELIOS levels in the nTreg and eTreg cells with (TGFβi) or without (NT) TGFβR1 inhibitor treatment for five weeks. This data is representative of experiments conducted in three donors. **(J)** Line charts summarizing the percentage of FOXP3^+^ population in nTreg and eTreg cells at indicated time points. Data are presented as Mean ± SEM. ^∗^p < 0.05, two-sided t test. n = 3 donors.

To investigate the role of TGFβ in different Treg subpopulations, we isolated human naïve (CD4^+^CD25^+^CD127^low^CD45RA^hi^CD45RO^low^) Treg cells and effector (CD4^+^CD25^+^CD127^low^CD45RA^low^CD45RO^hi^) Treg cells from human Leukopak samples by flow cytometry sorting (**Figure 1A**) and validated their purity post sorting (**Figure 1B**). The isolated naïve and effector Treg cells were activated and cultured with or without TGFβ signaling inhibition (TGFβi) by the small molecule inhibitor (SB-431542) that targets TGFβ receptor 1 (33). We find that naïve Treg cells and effector Treg cells are transcriptionally distinct from each other even without TGFβi treatment (**Figure 1C**). Intriguingly, TGFβi shifted the transcriptional state of naïve Treg cells toward effector Treg cells, while further polarizing effector Treg cells away from the major Treg colonies (**Figure 1C**). Gene set enrichment (GSEA) revealed that naïve Treg cells inherently exhibit higher TGFβ signaling activity upon expansion compared to effector Treg cells (**Figure 1D**, **Supplementary Figure S1A**). The increased TGFβ signaling is consistent with the higher expression of TGFβ receptor II in naïve Treg cells compared to effector Treg cells (**Supplementary Figure S1B**). We found that the higher expression of TGFβ receptor II also exists in freshly isolated naïve Treg cells (**Figure 1E**, **Supplementary Figures S1C, D**). These findings underscore the importance of TGFβ signaling in cell fate determination.

The loss of Treg cell identity is commonly associated with the gain of T helper (Th) cell function with increased proinflammatory cytokine production (10, 34). GSEA analysis revealed that TGFβ inhibition destabilized both Treg subsets by suppressing Treg signature genes and promoting proinflammatory pathways (**Figures 1F, G**, **Supplementary Figures S1E, F**). Specifically, TGFβ inhibition disrupted *FOXP3* RNA expression while increasing the expression of Th master regulators *TBX21* and *GATA3* (**Figure 1H**), as well as the expression of proinflammatory cytokines such as *IFNG*, *IL4, IL5, IL13* and *IL17A* (**Supplementary Figure S1E**). The TGFβ inhibition-induced Treg destabilization is more severe in effector Treg cells compared to those in naïve Treg cells, which is consistent with the knowledge that naïve Treg cells are more stable than effector Treg cells (4–6). This observation is further supported by the loss of FOXP3 protein expression and an increase in proinflammatory cytokine production, with effector Treg cells being more sensitive than naïve Treg cells (**Figures 1I, J**). Together, our findings highlight a critical role for TGFβ signaling in maintaining the stability of human Treg cells and suggest the level of TGFβ signaling might positively correlate with Treg stability in naïve and effector Treg subsets.

### 3.2 SKI expression negatively correlates with TGFβ signaling in Treg subsets

**Figure 2:**
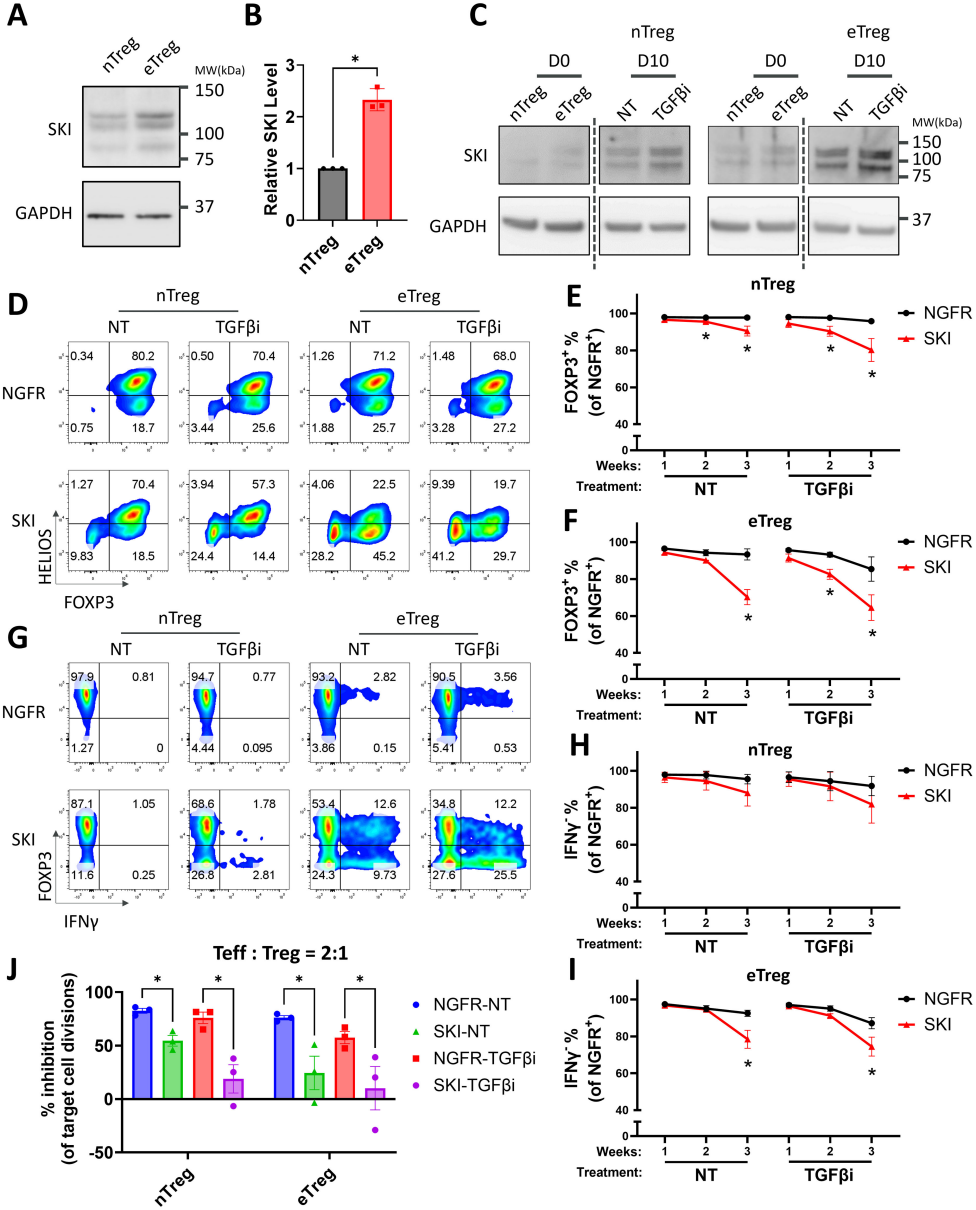
Overexpression of SKI destabilizes human Treg cells and further promotes TGFβi induced Treg destabilization. **(A)** The expression level of SKI protein in expanded naïve Treg (nTreg) cells and effector Treg (eTreg) cells analyzed by immunoblots with GAPDH as loading control. The blots are representative of three donors. Note: The endogenous SKI shows multiple bands due to post-translational modifications, a pattern previously reported before using the same antibody clone (38). **(B)** The expression level of SKI protein was quantitated and normalized to GAPDH to calculate normalized protein levels and then compared to nTreg cells to determine relative protein levels. Data are presented as Mean ± SEM. ^∗^p < 0.05, two-sided t test. n = 3 donors. **(C)** The expression level of SKI and GAPDH protein in naïve Treg cells treated with (TGFβi) or without (NT) TGFβR1 inhibitor for 0 and 10 days, analyzed by immunoblots. Blots from the same gels are separated by dotted lines. The blots are representative of three donors. **(D–I)** Expanded nTreg and eTreg cells transduced with NGFR or SKI-T2A-NGFR (SKI) were treated with (TGFβi) or without (NT) TGFβR1 inhibitor for three weeks. Levels of FOXP3, HELIOS, and IFNγ within the NGFR^+^ population were assessed weekly via flow cytometry. Representative flow cytometry profiles showing FOXP3 and HELIOS levels **(D)** and FOXP3 and IFNγ levels **(G)** are shown. The flow cytometry profiles are representative of experiments conducted in four donors. Line charts summarizing the percentage of FOXP3^+^ **(E, H)** and IFNγ^-^ cells **(H, I)** within the NGFR^+^ population in nTreg cells **(E, H)** and eTreg cells **(F, I)** at indicated time points are shown. Data are presented as Mean ± SEM. ^∗^p < 0.05, two-sided t test. n = 4 donors. **(J)** nTreg or eTreg cells carrying SKI or the empty vector (NGFR) were treated with (TGFβi) or without (NT) TGFβR1 inhibitor for three weeks and then subjected to an *in vitro* suppression assay. Treg cells were cultured with Teff cells at a 2:1 ratio (Treg: Teff) for four days. Following incubation, Teff proliferation was assessed by CTV dilution and flow cytometry. Suppression efficiency was calculated as “% inhibition of Teff division,” determined by the percentage reduction in the division index of Teff cells compared to Teff cells cultured alone. Data are presented as Mean ± SEM. ^∗^p < 0.05, two-sided t test. n = 3 donors.

We next sought to find the critical regulators of TGFβ signaling responsible for the stability of naïve Treg and effector Treg subsets. Previous studies have shown that SKI, a major negative regulator of the TGFβ signaling pathway, is crucial in the fate determination and functionality of mouse induced Treg (iTreg) and Th17 cells (17, 35–37). While SKI inhibits TGFβ downstream transcriptional factors like SMAD proteins, it can also be suppressed by TGFβ signaling via an E3 ligase ARKADIA (18). To test if TGFβ stabilizes human naturally occurring Treg cells through SKI regulation, we measured the protein levels of SKI in the two subpopulations of human Treg cells. We found that naïve Treg cells harbor a significantly lower amount of SKI protein compared with effector Treg cells (**Figures 2A–C**). However, we did not observe such a difference in the RNA level of SKI (**Supplementary Figure S1A**), indicating that the expression level of SKI is mainly regulated through post-translational modification (reviewed in (16)). Moreover, *in vitro* expansion increased the protein expression level of SKI in Treg cells, with a more pronounced upregulation observed in eTreg cells. As expected, blocking TGFβ signaling by TGFβi treatment further enriched SKI protein in those cells (**Figure 2C**). Importantly, this pattern of SKI upregulation closely parallels the trend of Treg destabilization observed during culture (**Figure 1J**). Our results indicate SKI protein expression levels reflect the differential TGFβ signaling in Treg subsets and is upregulated upon TGFβi treatment during Treg destabilization.

### 3.3 Ectopic expression of SKI destabilizes human Treg cells and impairs Treg suppressive function

To further investigate whether SKI is functionally relevant to Treg destabilization, we overexpressed SKI in Treg cells and monitored the stability of these cells in the presence or absence of TGFβ blockade. Notably, overexpression of SKI significantly decreased the levels of both FOXP3 and HELIOS-the latter widely considered a marker of stable human Treg cells (39) in both naïve Treg and effector Treg cells, with more severe Treg destabilization in effector Treg subsets (**Figures 2D–F**, **Supplementary Figures S2A, B**). Moreover, TGFβ inhibition further exacerbated Treg destabilization in both populations with SKI overexpression (**Figures 2D–F**, **Supplementary Figures S2A, B**). This additive effect of TGFβ inhibition is likely attributed to a further enhanced ectopic protein level by TGFβ inhibition (**Supplementary Figure S2C**). In line with our transcriptomic observations from TGFβi-treated Treg cells (**Supplementary Figure S1E**), SKI overexpression also increased the protein level of a key proinflammatory cytokines, IFNγ, with the effects being more pronounced in effector Treg cells (**Figures 2G–I**, **Supplementary Figures S2D, E**). In addition, we observed the presence of a FOXP3^+^IFNγ^+^ population upon treatment, especially in the effector Treg cells (**Figure 2G**). This phenotype suggests that TGFβ inhibition and SKI overexpression could induce Treg cell intrinsic inflammatory cytokine production before FOXP3 loss, highlighting the importance of Treg cell intrinsic TGFβ signaling for their stability.

We next assessed the immune suppressive function of these cells, using an *in vitro* suppression assay. Consistent with the loss of Treg cell identity (**Figures 2D–F**), SKI overexpression dramatically dampened the immune suppression ability of these Treg cells, with a more dramatic effect on that of effector Treg cells, indicating that effector Treg cells are more sensitive to TGFβi-induced Treg function loss compared to naïve Treg cells (**Figure 2J**, **Supplementary Figures S2F, G**). These findings demonstrate that SKI destabilizes Treg cells, and that SKI expression levels potentially correlate with Treg instability upon TGFβi treatment. These findings also suggest that the comparatively lower stability of effector Treg cells may be attributed to their elevated endogenous SKI levels compared to naïve Treg cells.

### 3.4 Degradation-resistant SKI further destabilizes Treg stability and functionality

**Figure 3:**
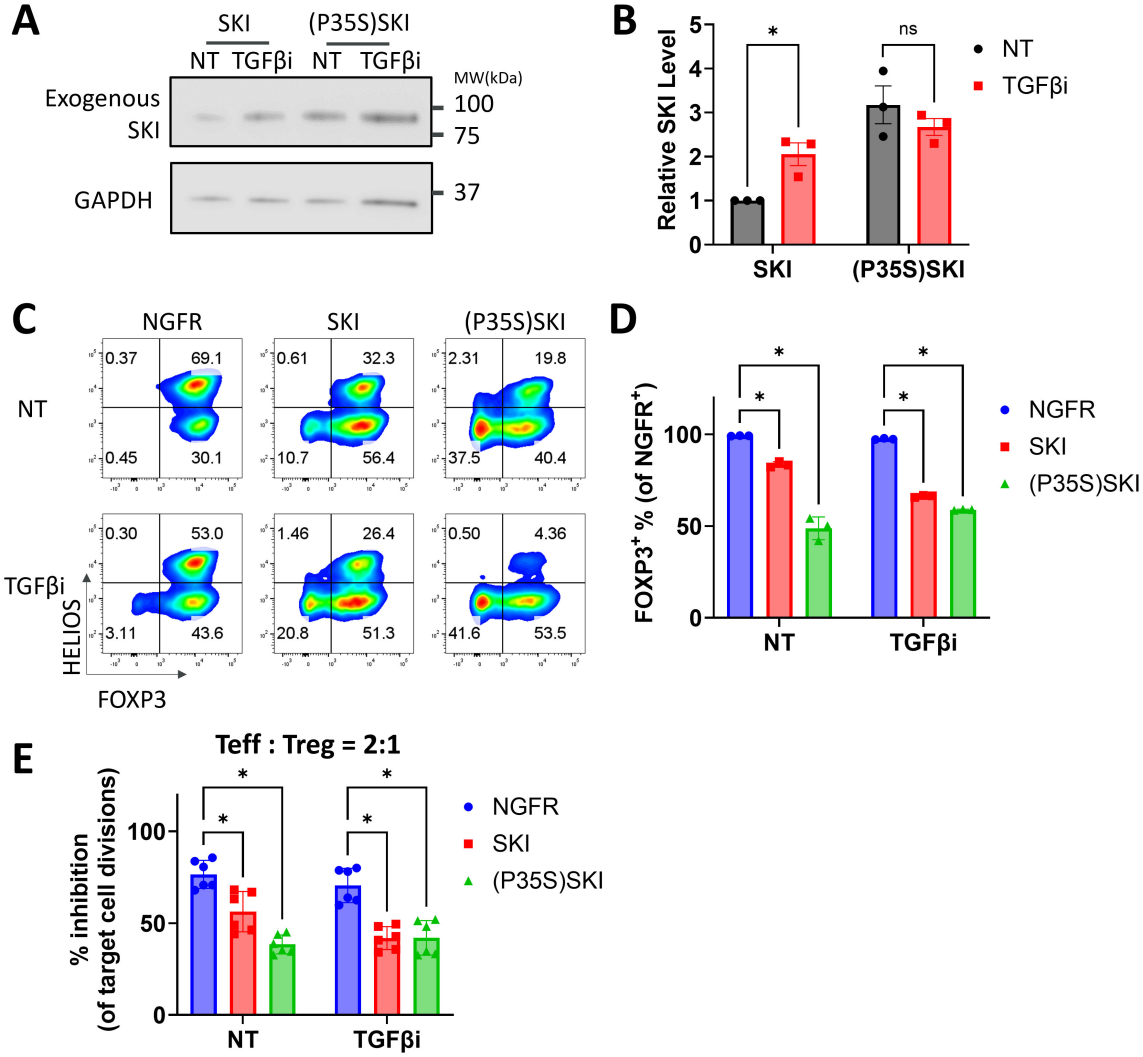
A degradation resistant SKI further destabilizes human Treg cells. **(A, B)** Naïve Treg cells transduced with SKI or (P35S) SKI were treated with (TGFβi) or without (NT) TGFβR1 inhibitor for three days, followed by immunoblots targeting SKI and GAPDH. **(A)** Representative immunoblots of three donors. **(B)** SKI levels were quantitated and normalized to GAPDH to calculate normalized protein levels and then compared to the NT-SKI samples to determine relative protein levels. Data are presented as Mean ± SD. ^∗^p < 0.05, two-sided t test. n = 3 donors. **(C-E)** Naïve Treg cells transduced with NGFR or SKI-T2A-NGFR (SKI) or (P35S)SKI-T2A-NGFR ((P35S)SKI) were treated with (TGFβi) or without (NT) TGFβR1 inhibitor for three weeks. **(C)** Levels of FOXP3 and HELIOS within the NGFR^+^ population were assessed via flow cytometry. Representative flow cytometry profiles showing FOXP3 and HELIOS levels. n = 3 donors. **(D)** A bar graph summarizing the level of FOXP3 within the NGFR^+^ population. Data are presented as Mean ± SD. ^∗^p < 0.05, two-sided t test. Data present n = 3 technical replicates from a single donor. This experiment was independently repeated with cells from three donors, yielding similar results. **(E)** These Treg cells were then subjected to an *in vitro* suppression assay and cultured with Teff cells at a 2:1 ratio (Treg: Teff) for four days. Following incubation, Teff proliferation was assessed by CTV dilution and flow cytometry. Suppression efficiency was calculated as “% inhibition of Teff division,” determined by the percentage reduction in the division index of Teff cells compared to Teff cells cultured alone. Data are presented as Mean ± SD. ^∗^p < 0.05, two-sided t test. n = 6 replicates from two donors.

It has been well-established that TGFβ regulates SKI through protein degradation (reviewed in (16)). We found that TGFβi stabilizes SKI protein in Treg cells while further disrupting Treg stability in the combination of SKI overexpression. To further evaluate the role of TGFβ-mediated SKI degradation in Treg stability, we generated a mutant of SKI (P35S) that is resistant to TGFβ-induced degradation (40). The SKI mutant was verified in naïve Treg cells, where its protein level remained steady regardless of the presence of TGFβi. In contrast, the wildtype SKI protein level was significantly increased by TGFβ inhibition (**Figures 3A, B**). Functionally, the mutated SKI was more potent in destabilizing Treg cells compared to the wildtype SKI. Its destabilization effect could not be further increased upon TGFβ inhibition, as measured by decreased FOXP3 level (**Figures 3C, D**) and impaired suppression ability (**Figure 3E**, **Supplementary Figure S3**). Therefore, our findings demonstrated that the expression of SKI drives the Treg cell destabilization and the degradation of SKI by TGFβ signaling is essential to maintain Treg stability and functionality.

### 3.5 SKI destabilizes human Treg cells by suppressing Treg signature genes and promoting pro-inflammatory pathways

**Figure 4:**
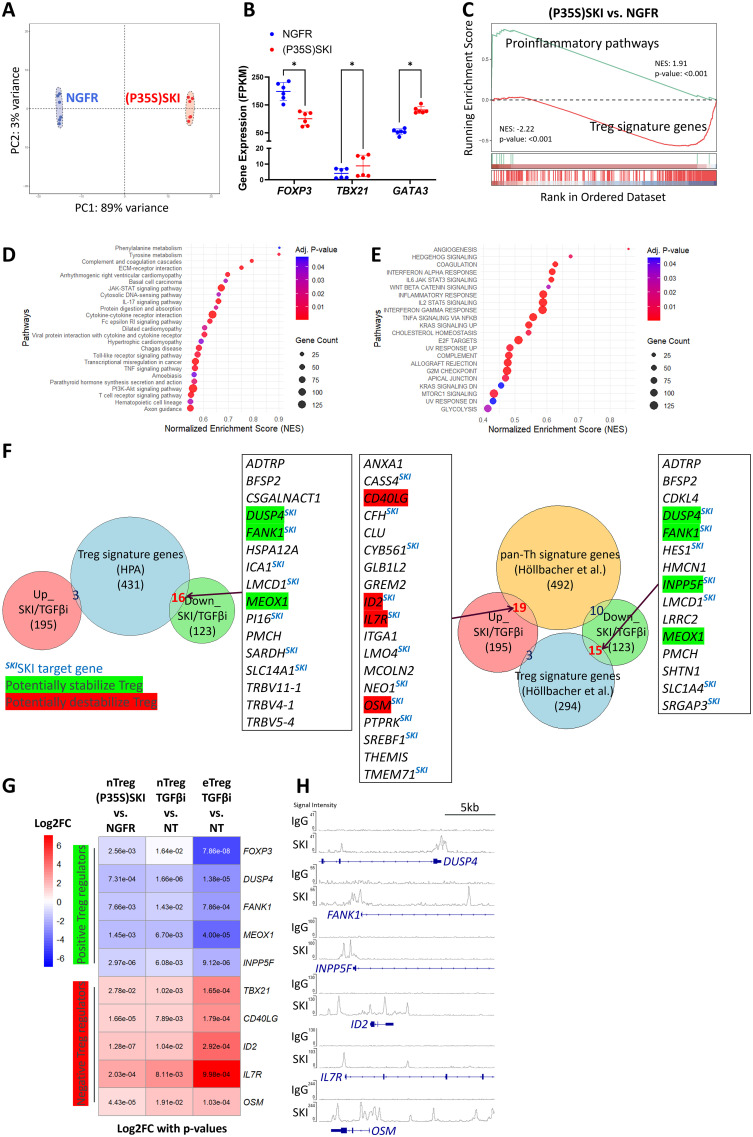
Upregulation of SKI transcriptionally disrupts Treg cell identity. **(A)** Principal Component Analysis (PCA) of whole genome transcriptome data in naïve Treg cells transduced with NGFR or (P35S)SKI. n = 6 samples from two donors. **(B)** A scatter plot illustrates gene expression levels of the indicated genes in naïve Treg cells transduced with NGFR or (P35S)SKI. Data are presented as Mean ± SD. ^∗^p < 0.05, two-sided t test. n = 6 samples from two donors. **(C)** Gene set enrichment analysis (GSEA) was performed on the indicated sample groups and gene collections. Normalized Enrichment Scores (NES) and p-values were calculated using permutation tests and are labeled accordingly. **(D, E)** KEGG **(D)** and MSigDB Hallmarks **(E)** enrichment analysis for differently expressed genes (DEGs) ((P35S)SKI vs. NGFR, adjusted p<0.05,|FC|>2) using the GSEA method. **(F)** Overlap analysis of commonly up- and down-regulated genes by (P35S)SKI overexpression and TGFβi in Treg cells (Up_SKI/TGFβi and Down_SKI/TGFβi, shared Differentially Expressed Genes across three RNA-seqs, adjusted p < 0.05, |FC| > 2) with gene sets of Treg signature genes (41) (left panel) or with Th/Treg signature genes (42) (Right panel). Important overlapping genes are listed, with known positive regulators of Treg cells highlighted in green and negative regulators in red. SKI target genes are also noted. **(G)** A heatmap illustrating important Up_SKI/TGFβi and Down_SKI/TGFβi, which are known to modulate Treg identity or functionality. Log2FC values are depicted by colors, with p values labeled. **(H)** CHIP-seq profiles showing SKI occupancy on the indicated genes. Data were obtained from GSE107556 and visualized using the IGV browser.

To investigate how SKI regulates Treg stability and functionality, we overexpressed the SKI mutant (SKI (P35S)) in Treg cells and evaluated the global transcriptome changes by mRNA-sequencing. We chose the degradation resistant SKI mutant as it can minimize the effect of TGFβ-mediated downregulation of SKI (**Figure 3**). We found that the ectopic expression of the SKI mutant significantly altered the Treg transcriptome (**Figure 4A**), revealing 1788 differentially expressed genes (**Supplementary Figure S4A**). The results aligned with the knowledge that the target genes of SKI/SNON such as SKIL (43) and SMAD7 (44) appeared as top downregulated genes by mutant SKI overexpression (**Supplementary Figure S4A**). The transcriptional level data also aligned with the protein level data from both wildtype (**Figures 2D–I**) and mutant SKI (**Figures 3C, D**). SKI mutant expression destabilizes naïve Treg cells by reducing Treg signature genes expression, including *FOXP3*, while increasing the expression of Th master regulators *TBX21* and *GATA3*, as well as pro-inflammatory cytokines (**Figures 4B, C**). Furthermore, we observed that SKI influences the secretion and signaling of pro-inflammatory cytokines through unsupervised GSEA analysis using KEGG and MSigDB_Hallmarks data sets. Notably, pathways mediated by IL17, IL6, and IFN were enhanced (**Figures 4D, E**). Additionally, SKI activated pathways that undermine Treg stability and functionality. These pathways include the PI3K-AKT-mTORC pathway, which influences Treg vs. Teff lineage commitment through metabolic regulations (45, 46), the WNT pathway, which disrupts FOXP3 transcriptional activity (47), and glycolysis, whose unrestrained upregulation impairs Treg function (48).

### 3.6 TGFβ-SKI axis regulates key target genes responsible for Treg to effector T cells conversion

We aimed to determine the most significant components causing Treg destabilization that are commonly regulated by TGFβ-SKI axis. Since TGFβ-inhibition-increased endogenous SKI and overexpressed SKI both destabilized Treg cells and disrupted Treg functionality (**Figure 2**), we overlapped differentially expressed genes (DEGs) from mutant SKI overexpression in naïve Treg cells with those resulting from TGFβ inhibition in both naïve Treg and effector Treg cells (**Supplementary Figures S4B, C**). This analysis yielded 195 upregulated and 123 downregulated genes. By comparing these DEGs with Treg signatures defined by the Human Protein Atlas (41), we found 16 Treg signature genes downregulated and 3 regulated (**Figure 4F** left panel), supporting our GSEA findings that SKI overexpression (**Figure 4C**) and TGFβ inhibition (**Figures 1F, G**) disrupt Treg identity. A similar analysis using a recently defined pan-Th versus Treg signature gene set (42) revealed 15 downregulated Treg signature genes and 19 upregulated Th signature genes (**Figure 4F** right panel). Among DEGs tied to Treg or Teff signatures, we found key genes critical for Treg function were downregulated, including *DUSP4* (49), *FANK1* (50, 51), *MEOX1* (52), and *INPP5F* (53, 54). In contrast, we found that Treg negative regulators like *ID2* (55), *IL7R* (56) and pro-inflammatory molecules *CD40LG* (57) and *OSM* (58) were upregulated. These genes indicate the shift from Treg to Teff facilitated by SKI or TGFβ inhibition, detailed in (**Figure 4G**). Although *FOXP3*, the Treg master regulator, was not prominent in our analysis due to a modest fold change (|FC| < 2) under TGFβ inhibition (**Figure 1G**), it remains part of our list. Similarly, *TBX21*, the Th1 master regulator, consistently upregulated by both SKI (**Figure 4B**) and TGFβi (**Figure 1G**), was included due to its relevance despite not being in the pan-Th signature genes (42). SKI is known as a transcriptional coregulator functioning in either the activation or repression of its target genes (17). In line with this, we identified several genes from our list, including *DUSP4*, *FANK1*, *INPP5F*, *ID2*, *IL7R*, and *OSM*, as potential SKI targets due to SKI’s known occupation of their regulatory domains (59, 60) (**Figure 4H**). Collectively, TGFβ-SKI signaling regulates Treg stability by regulating multiple signaling and metabolic pathways that are responsible for Treg to effector T cells conversion.

### 3.7 E3 ubiquitin ligase ARKADIA protects human Treg cells stability by degrading SKI

**Figure 5:**
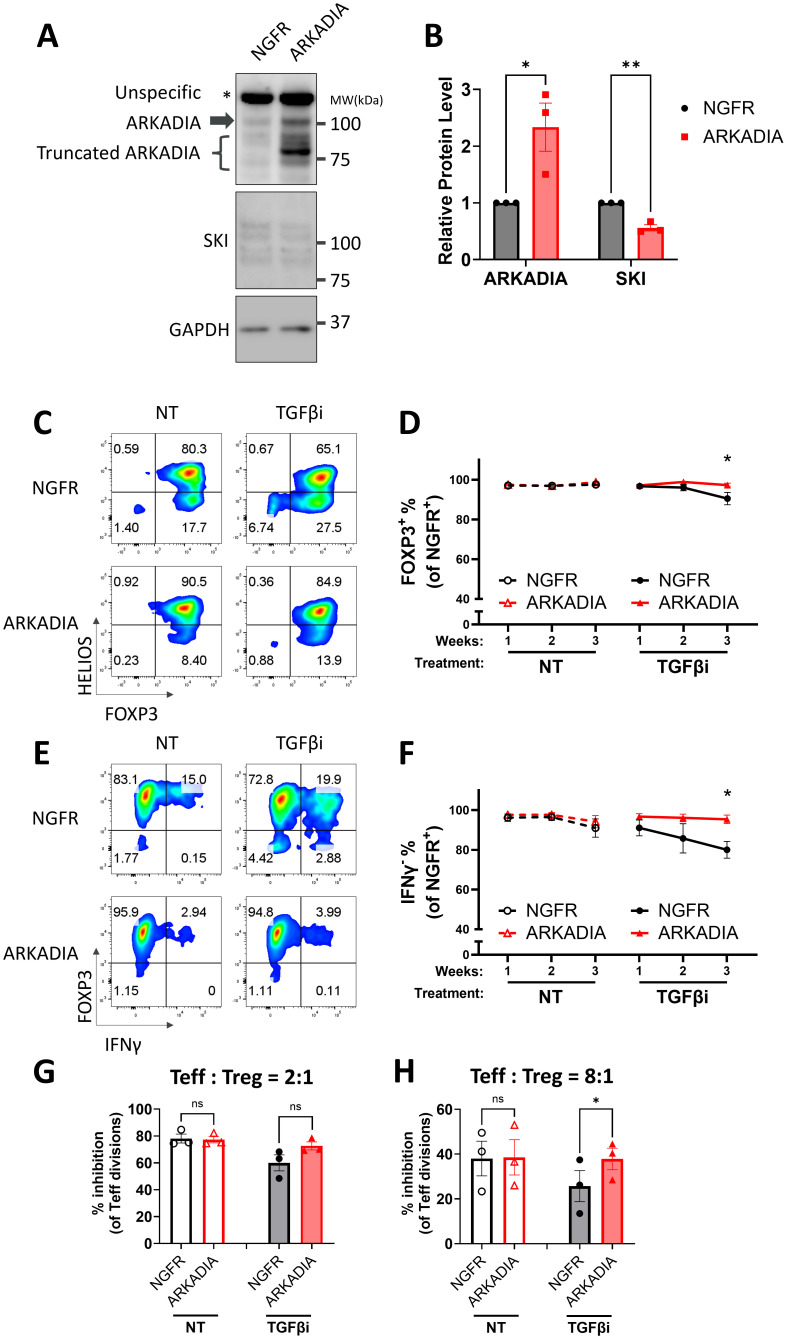
ARKADIA prevents Treg destabilization upon TGFβ inhibition. **(A, B)** Naïve Treg cells carrying NGFR or ARKADIA-T2A-NGFR (ARKADIA) were cultured for seven days, followed by immunoblot targeting AKADIA, SKI, and GAPDH. **(A)** Representative immunoblots of three donors. Note: ARKADIA shows multiple bands due to truncation, a pattern previously reported using the same antibody clone (20). **(B)** ARKADIA and SKI levels were quantitated and normalized to GAPDH to calculate normalized protein levels and then compared to the NGFR samples to determine relative protein levels. Data are presented as Mean ± SEM. ^∗^p < 0.05, two-sided t test. n = 3 donors. **(C-H)** Naïve Treg cells carrying NGFR or ARKADIA-T2A-NGFR (ARKADIA) were treated with (TGFβi) or without (NT) TGFβR1 inhibitor for three weeks. Levels of indicated proteins within the NGFR^+^ population were assessed weekly via flow cytometry. Representative flow cytometry profiles showing FOXP3 and HELIOS levels **(C)** or FOXP3 and IFNγ levels **(E)** in transduced Treg cells after three weeks of treatment. The flow cytometry profiles are representative of experiments conducted in three donors. Line charts summarize the percentage of FOXP3^+^ cells **(D)** or IFNγ^-^ cells **(F)** within the NGFR^+^ population in the Treg cells. Data are presented as Mean ± SEM. ^∗^p < 0.05, two-sided t test. n = 3 donors. After the three-week treatment, those Treg cells were subjected to an *in vitro* suppression assay by being cultured with Teff cells at a 2:1 ratio (Treg: Teff) **(G)** or 8:1 ratio (Treg: Teff) **(H)** for four days. Following incubation, Teff proliferation was assessed by CTV dilution and flow cytometry. Suppression efficiency was calculated as “% inhibition of Teff division,” determined by the percentage reduction in the division index of Teff cells compared to Teff cells cultured alone. Data are presented as Mean ± SEM. ^∗^p < 0.05, two-sided t test. n = 3 donors.

It is well defined that TGFβ signaling degrades SKI and its analog SNON via the E3 ubiquitin ligase ARKADIA in T cells as well as other cell types (18, 36, 61). Specifically, it has been reported that Arkadia degrades Ski and SnoN to enhance TGFβ-induced Treg differentiation from naïve CD4 T cells in mouse models (36). Another study further demonstrated that both SKI and SNON are targets of ARKADIA in response to TGFβ (20). We also observed the functional redundancy of SKI and SNON (36), as single knockout of SKI failed to prevent TGFβi induced Treg destabilization (data not shown). We hypothesized that TGFβ degrades SKI via ARKADIA to stabilize Treg cells. We thus overexpressed ARKADIA in naïve Treg cells and confirmed the following degradation of SKI (**Figures 5A, B**). We then evaluated its effect on Treg stability with or without TGFβ inhibition and found that overexpression of ARKADIA prevented the FOXP3 loss and prevented the production of IFNγ in naïve Treg cells under TGFβi treatment (**Figures 5C–F**). Moreover, overexpression of ARKADIA preserved the Treg immune suppressive function in naïve Treg cells with TGFβi treatment (**Figures 5G–H**, **Supplementary Figure S5**).

**Figure 6:**
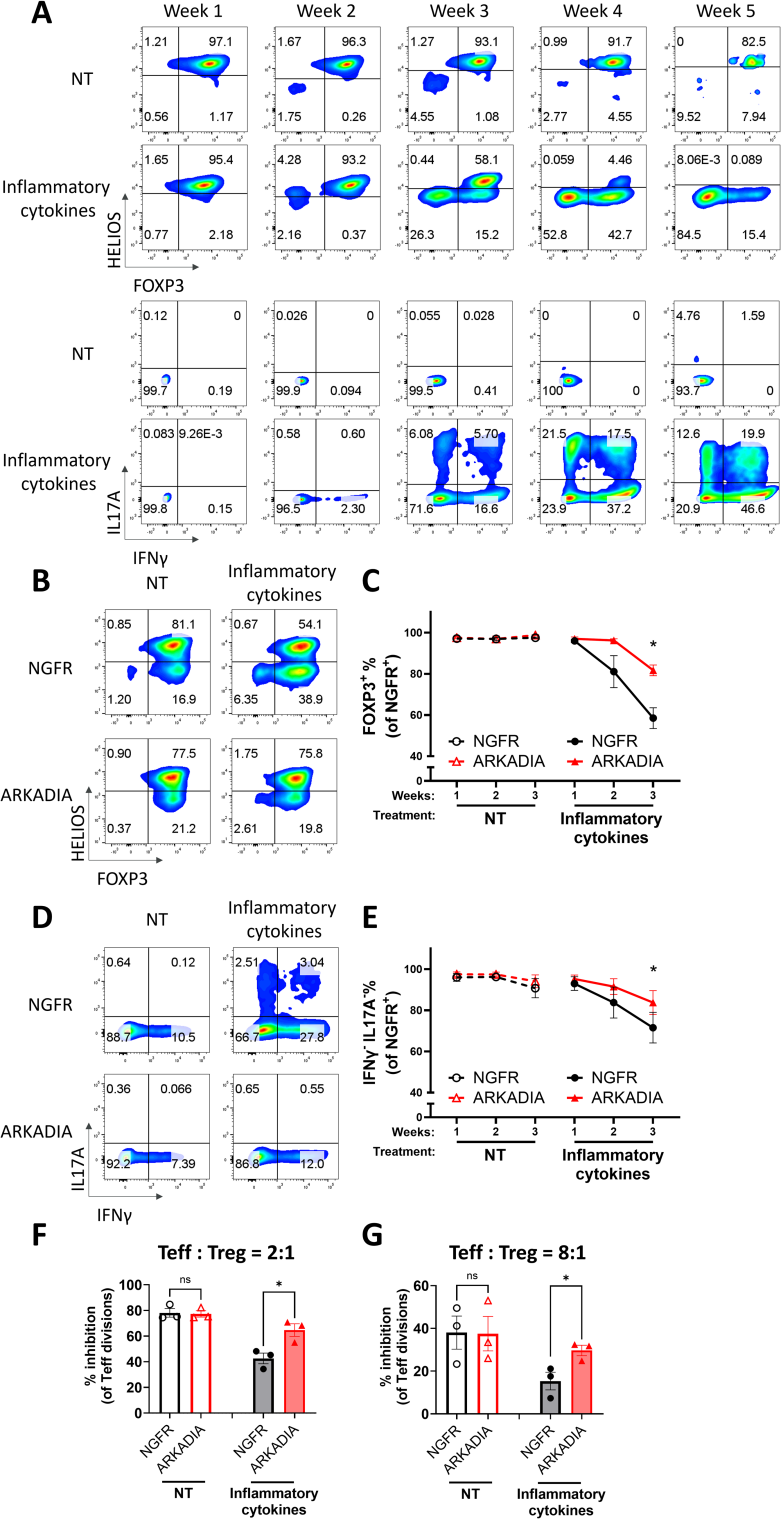
ARKADIA prevents Treg destabilization and preserves Treg function in the presence of inflammatory stimulation. **(A)** Naïve Treg cells were treated with (inflammatory cytokines) or without (NT) pro-inflammatory cytokines for five weeks. FOXP3, HELIOS, IL17A and IFNγ levels were assessed weekly via flow cytometry. The flow cytometry profiles are representative of experiments conducted in more than three donors. **(B–G)** Naïve Treg cells transduced with NGFR or ARKADIA-T2A-NGFR (ARKADIA) were treated with (inflammatory cytokines) or without (NT) pro-inflammatory cytokines for three weeks. Levels of indicated proteins within the NGFR^+^ population were assessed weekly via flow cytometry. Representative flow cytometry profiles showing FOXP3 and HELIOS levels **(B)** or IL17A and IFNγ levels **(D)** in transduced Treg cells after three weeks of treatment. The flow cytometry profiles are representative of experiments conducted in three donors. Line charts summarize the percentage of FOXP3^+^ cells **(C)** or IFNγ^-^IL17A^-^ cells **(E)** within the NGFR^+^ population in the Treg cells. Data are presented as Mean ± SEM. ^∗^p < 0.05, two-sided t test. n = 3 donors. After the three-week treatment, those nTreg cells were subjected to an *in vitro* suppression assay, by being cultured with Teff cells at a 2:1 ratio (Treg: Teff) **(F)** or 8:1 ratio (Treg: Teff) **(G)** for four days. Following incubation, Teff proliferation was assessed by CTV dilution and flow cytometry. Suppression efficiency was calculated as “% inhibition of Teff division,” determined by the percentage reduction in the division index of Teff cells compared to Teff cells cultured alone. Data are presented as Mean ± SEM. ^∗^p < 0.05, two-sided t test. n = 3 donors.

In autoimmune patients, chronic inflammation could destabilize Treg cells and disrupt their immunosuppressive function (10, 62, 63). Since inflammatory cytokines could suppress TGFβ signaling (64), we hypothesized that ARKADIA could block inflammatory-cytokine-induced Treg destabilization as well. To do so, we employed an inflammatory condition to destabilize Treg cells with or without ARKADIA overexpression. We found the inflammatory cytokine combination of IL-6, IL-1β and IL-23 destabilized naïve Treg cells and converted them into Th1- and Th17- like cells (**Figure 6A**). ARKADIA overexpression significantly prevented the FOXP3 loss and suppressed the production of IFNγ and IL-17A cytokines in naïve Treg cells (**Figures 6B–E**), underscoring its ability to maintain Treg identity even under inflammatory conditions. Importantly, overexpression of ARKADIA notably preserved the suppressive ability of naïve Treg cells in response to inflammatory stimuli (**Figures 6F, G**, **Supplementary Figure S5**). In summary, our data suggests that ARKADIA is essential to prevent Treg destabilization and engineering ARKADIA has the potential to enhance Treg cell stability and functionality.

## 4 Discussion

Treg cells play an essential role in the maintenance of immune homeostasis and tolerance. Their unique characteristics, heterogeneity and plasticity, are critical for adapting to distinct inflammatory cues while retaining their suppressive capacity and effectively managing different types of inflammatory scenarios (13). Meanwhile, Treg cells must maintain their identity and functionality to prevent acquiring pathogenic phenotype. This is achieved via mechanisms like epigenetic remodeling to promote FOXP3 stability and regulation of their capacity to sense inflammatory cytokines, primarily by modulating cytokine receptors and intracellular regulators (65). The TGFβ signaling is not only instrumental for induction of Treg cells from naïve CD4 T cells, but also regulates the thymic development of natural Treg cells (14). However, it remains unclear whether and how TGFβ signaling contributes to the stability of established Treg cells. In this study, we demonstrate that the blockade of TGFβ signaling destabilizes human Treg cells and converts them into Th-like cells expressing pro-inflammatory cytokines. Furthermore, we show that the TGFβ signaling is differentially regulated between naïve Treg cells and effector Treg cells, and that naïve Treg cells are more stable upon TGFβ blockade treatment compared to that of effector Treg cells. Mechanically, we show that TGFβ regulates human Treg stability through a TGFβ-ARKADIA-SKI axis. By leveraging this pathway, we have developed novel strategies to stabilize human naïve Treg cells by overexpressing ARKADIA, which enhances Treg stability even under inflammatory conditions. Overall, this study broadens our understanding of the molecular regulation of human Treg cell stability and functionality.

While effector Treg cells are generally considered to have enhanced suppression against specific conventional T cells—mediated by the adoptive expression of corresponding transcription factors (e.g., TBET^+^ Treg cells controlling Th1 cells, GATA3^+^ Treg cells controlling Th2 cells, and RORγT^+^ Treg cells controlling Th17 cells) (8)—effector Treg cells are typically less stable than naïve Treg cells, presenting challenges for Treg cell therapies (4–6). Our research shows that TGFβ dependency for stability is more significant in human effector Treg cells (CD45RO^+^) than in naïve Treg cells (CD45RO^-^), potentially due to higher SKI protein levels. Aligning with this, we provide evidence that ectopic SKI expression increases TGFβ dependency in both naïve Treg and effector Treg cells. Additionally, our transcriptome analysis reveals an intrinsic reduction of TGFβ signaling in effector Treg cells, which we preliminarily attribute to a lower *TGFBR2* RNA level, a pattern consistent across multiple datasets. This also aligns with findings that TCR stimulation reduces TGFBR1 protein levels, affecting TGFβ signaling, despite reductions in RNA levels for both *TGFBR1* and *TGFBR2* (66). Our findings indicate that TGFβ signaling is differentially regulated in Treg subsets and the increased TGFβ signaling in naïve Treg cells contributes to enhanced stability.

In this work, we explore the mechanisms of human Treg regulation by SKI and TGFβ, by examining transcriptome changes following TGFβ inhibition and SKI overexpression in Treg cells. We anticipate that both modulations, which increase SKI levels in human Treg cells, would reveal common patterns of transcriptional regulation that could potentially disturb Treg stability. By overlapping differentially expressed genes (DEGs) from both approaches across naïve Treg and effector Treg cells, we found a significant proportion of shared DEGs, particularly in TGFβ-inhibited naïve Treg samples, which exhibited the mildest transcriptomic disturbance (195/425 in Up_DEGs and 123/365 in Down_DEGs). In this study, we focused on these common DEGs to identify genes known to regulate Treg cells and targeted by SKI. This approach allowed us to pinpoint several key target genes regulated by SKI. It is also important to note the existence of SMAD-/SKI- independent noncanonical signaling pathways triggered by TGFβ ligand (15), as well as TGFβ-/SMAD- independent signaling modulated by SKI (67, 68). These pathways might explain the large number of unique DEGs in TGFβ-inhibited effector Treg samples (724/1303 in Up_DEGs and 1525/2186 in Down_DEGs) and the mutant SKI-overexpressed naïve Treg samples (363/850 in Up_DEGs and 387/961 in Down_DEGs), respectively. Therefore, we cannot rule out the possibility that additional mechanisms might contribute to the TGFβi-mediated and SKI overexpression-mediated Treg destabilization. In summary, our transcriptional analysis has identified a novel role of SKI-driven transcriptional regulation of the stability and functionality of human Treg cells. We believe these findings lay the groundwork for future, more detailed mechanistic investigations into this axis.

TGFβ is central to Treg not only because Treg cells are among the primary producers of latent TGFβ1 and possess specific activation mechanisms, but also due to their high surface expression of the TGFβ receptor (69). At the molecular level, TGFβ signaling—particularly through SMAD3—can directly enhance FOXP3 expression by binding to the conserved non-coding sequence CNS1 within the FOXP3 locus (70). While this axis is essential for the generation of peripherally-derived Treg (pTreg) cells (71, 72) and iTreg cells (21, 22), but largely dispensable for thymus-derived (tTreg) cell generation under non-inflammatory conditions (73, 74). However, the role of TGFβ in the maintenance of Treg cells, especially regarding sustained FOXP3 expression, remains controversial, with prior studies in mouse models reporting mixed results (25–28). HELIOS cooperates with FOXP3 to reinforce the functionality and stability of Tregs and is widely recognized as a stable maker of Treg (39, 75, 76). Intriguingly, here we observed that SKI overexpression not only interrupts FOXP3 but also HELIOS levels in Treg cells. This observation is consistent with what others have observed in mice where Arkadia deficiency in Cd4 T cells increases the percentage of Helios^+^ Treg cells (36). The regulation of both FOXP3 and HELIOS by the TGFβ-ARKADIA-SKI axis highlights their central roles in maintaining human Treg stability and underscores the need for further investigation into this pathway.

Ski has been previously shown to prevent the differentiation of Th17 cells from naïve CD4 T cells in *in vitro* Th17 polarizing cytokine conditions (35, 37). In our study, we found that destabilization of SKI through ectopic ARKADIA overexpression protected human Treg cells from transitioning into a Th1-/Th17-like phenotype under similar conditions. This discrepancy may arise from differences in the regulatory mechanisms governing Th17 differentiation from Treg cells versus naïve CD4 T cells, as well as from variations in these mechanisms between humans and mice. Additionally, it may be attributed to the dose-dependent regulation by SKI (16), where ARKADIA overexpression only destabilizes the inherently low-dose endogenous SKI in Treg, which is insufficient to modulate Th17 differentiation from Treg cells. This discrepancy highlights the need for further investigation into the distinct signaling contexts under which TGFβ and SKI operate within human immune cells. Another limitation remains as our findings are primarily based on *in vitro* experiments. As we move forward, integrating insights from *in vitro* studies with *in vivo* experimentation to evaluate SKI-mediated Treg destabilization, and especially ARKADIA-mediated Treg stabilization, will further benefit our knowledge in Treg biology and Treg cell therapies.

Treg cell-based therapies are actively investigated to treat autoimmune diseases, organ transplantation rejection and GvHD (12). Techniques such as Chimeric Antigen Receptor (CAR) and antigen specific TCR are employed to enhance the specificity and potency of these therapies. However, a critical concern in using Treg cells as modality is their stability, defined by the stable maintenance of FOXP3 expression and suppressive activity, without the production of pro-inflammatory cytokines. While the stable FOXP3 expression has traditionally been viewed as the primary determinant of Treg stability, it is now clear that additional mechanisms play important roles, such as epigenetic modifications, metabolism changes and genetics factors (12).

Our investigation into the TGFβ-ARKADIA-SKI axis highlights a previously underappreciated mechanism governing the stability of naturally occurring human Treg cells—the major population used in Treg and CAR-Treg cell therapy clinical trials. We demonstrate for the first time in primary human Treg cells that TGFβ regulates SKI protein stability via the E3 ligase ARKADIA, with SKI negatively impacting Treg functionality. Though important differences exist between human and murine Treg biology and immune responses (77, 78), our findings extend and validate observations from murine models, in which Arkadia is required for murine Treg cell differentiation and the control of microbiota-induced mucosal inflammation (36). Importantly, by confirming this evolutionarily conserved pathway specifically in human natural Tregs—not just murine Treg cells and murine inflammation models—our work directly addresses a critical question in the field and provides mechanistic and translational insights with direct application for Treg cell-based therapies. Furthermore, we provide the first evidence that ARKADIA overexpression can enhance the stability of human Tregs in a cell-therapy-compatible setting, supporting its future exploration as a molecular target for improving Treg-based immunotherapies in inflammatory diseases.

In summary, our study uncovers a novel role of TGFβ-ARKADIA-SKI axis in human Treg cell stability and functionality. It also provides potential targets to either limit Treg stability to improve immunotherapies for tumor or enhance Treg stability of Treg cell therapies for inflammatory diseases.

## Data Availability

The datasets presented in this study can be found in online repositories. The names of the repository/repositories and accession number(s) can be found in the article/**Supplementary Material**.
