# Supplementary material for: Differential regulation of Treg stability in human naïve and effector Treg subsets by TGFβ-signaling via ARKADIA-SKI axis
Source: Front Immunol. 2025 Sep 9;16:1636434. doi: 10.3389/fimmu.2025.1636434 (PMC12454061; doi:10.3389/fimmu.2025.1636434)
Supplement: Supplementary file 3 [file DataSheet1.docx]

Supplementary Material

## Supplementary Figures


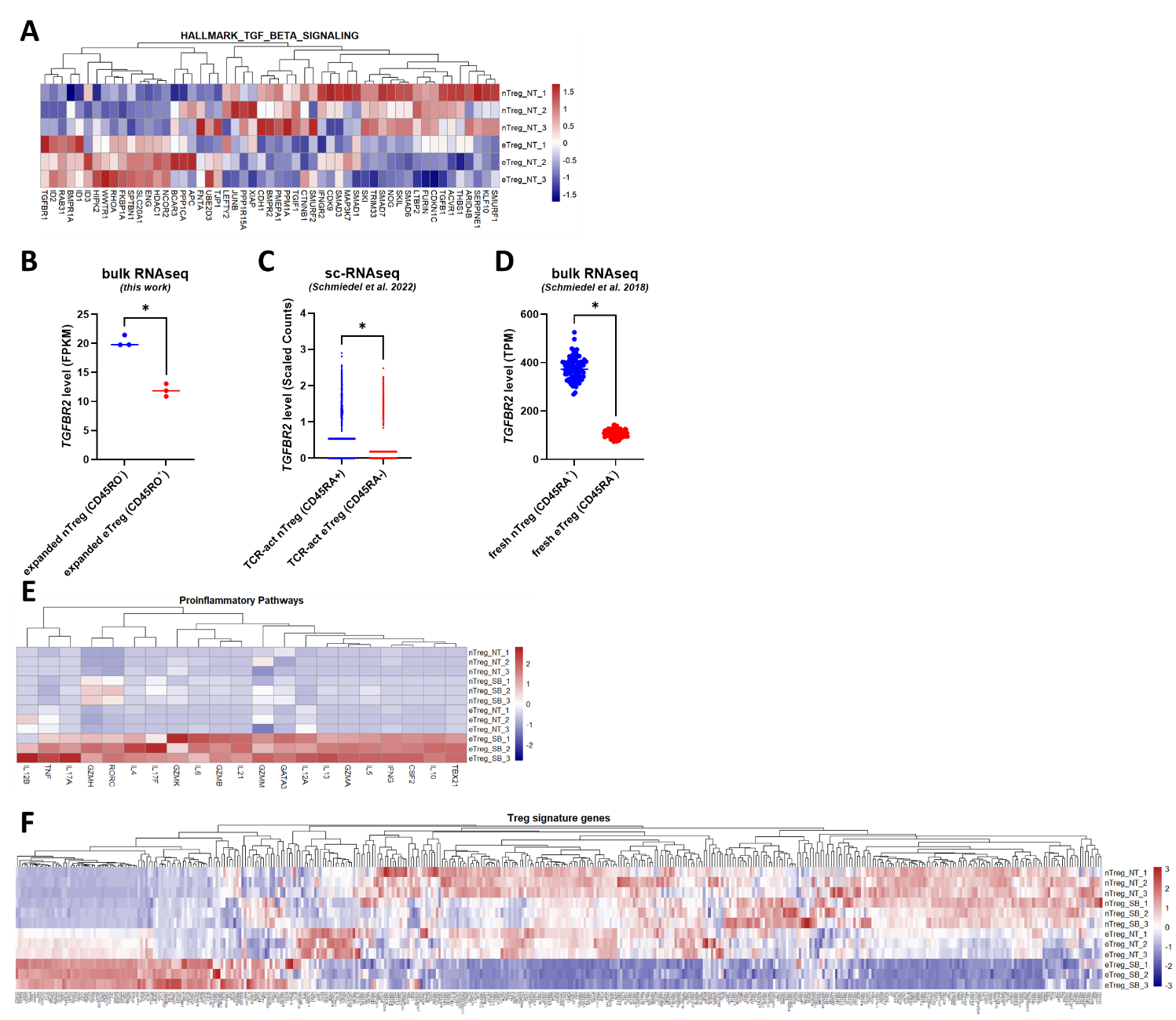


**Supplementary Figure 1.** **TGFβ signaling blockade destabilizes human natural occurring Treg cells. (A, F and G)** Freshly isolated naïve Treg (nTreg) cells and effector Treg (eTreg) cells were treated with (TGFβi) or without (NT) TGFβR1 inhibitor for five weeks, followed by RNA-seq analysis. Heatmaps of genes from Hallmark TGFβ signaling **(A)**, proinflammatory pathways **(F)**, and Treg signature genes **(G)** are shown. n = 3 replicates. **(B-D)** Scatter plots summarizing *TGFBR2* RNA expression measured by bulk RNA-seq or single cell RNA-seq in the indicated study across nTreg and eTreg. Data are presented as Mean ± SD. ^∗^p < 0.05, two-sided t test.


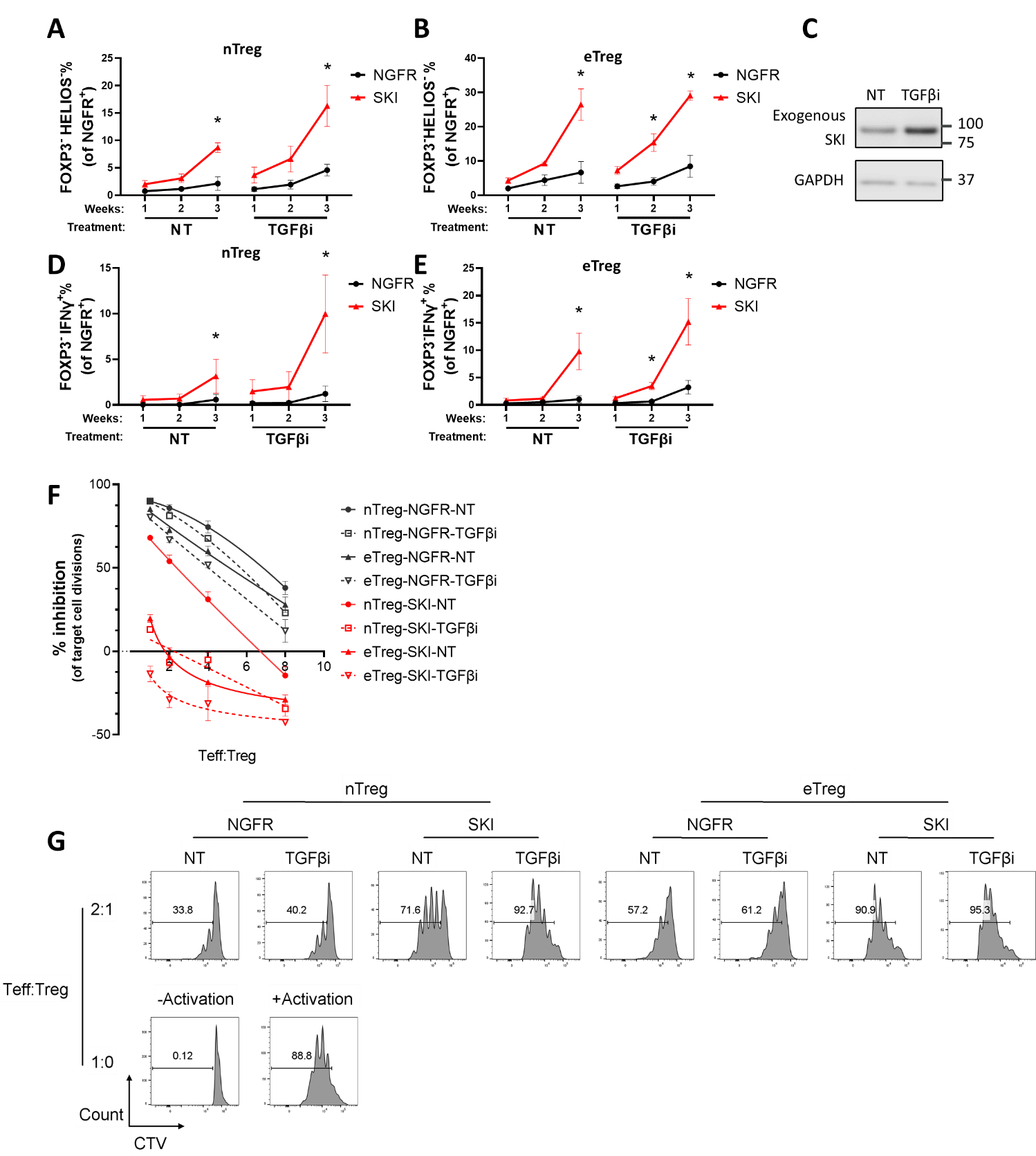


**Supplementary Figure 2.** **SKI destabilizes human naturally occurring Treg cells.** Expanded naïve Treg (nTreg) cells and effector Treg (eTreg) cells transduced with NGFR or SKI-T2A-NGFR (SKI) were treated with (TGFβi) or without (NT) TGFβR1 inhibitor for three weeks. **(A and B)** Line charts summarizing the percentage of FOXP3^-^HELIOS^-^ within the NGFR^+^ population of nTreg **(A)** and eTreg **(B),** assessed at indicated time points via flow cytometry**.** Data are presented as Mean ± SEM. ^∗^p < 0.05, two-sided t test. n = 4 donors.  **(C)** The expression level of SKI and GAPDH protein in nTreg cells carrying SKI treated with (TGFβi) or without (NT) TGFβR1 inhibitor for 3 days was analyzed by immunoblots. The blots are representative of 3 donors. Exogenous SKI shows a strong single band around the size of unmodulated SKI (75-100kDa) under low exposure. **(D and E)** Line charts summarizing the percentage of FOXP3^-^IFNγ within the NGFR^+^ population of nTreg **(D)** and eTreg **(E),** assessed at indicated time points via flow cytometry**.** Data are presented as Mean ± SEM. ^∗^p < 0.05, two-sided t test. n = 4 donors. **(F)** After the three-week treatment, these Treg cells were then subjected to an *in vitro* suppression assay. Treg cells were cultured with Teff cells at the indicated ratios (Treg:Teff) for four days. Following incubation, Teff proliferation was assessed by CTV dilution and flow cytometry. Suppression efficiency was calculated as “% inhibition of Teff division,” determined by the percentage reduction in the division index of Teff cells compared to Teff cells cultured alone. Data are presented as Mean ± SD. n = 3 replicates from one donor. ^∗^p < 0.05, two-sided t test. This setup is representative of experiments conducted in two donors. **(G)** A representative CTV profile of Teff cells. This setup is representative of experiments conducted in three donors.


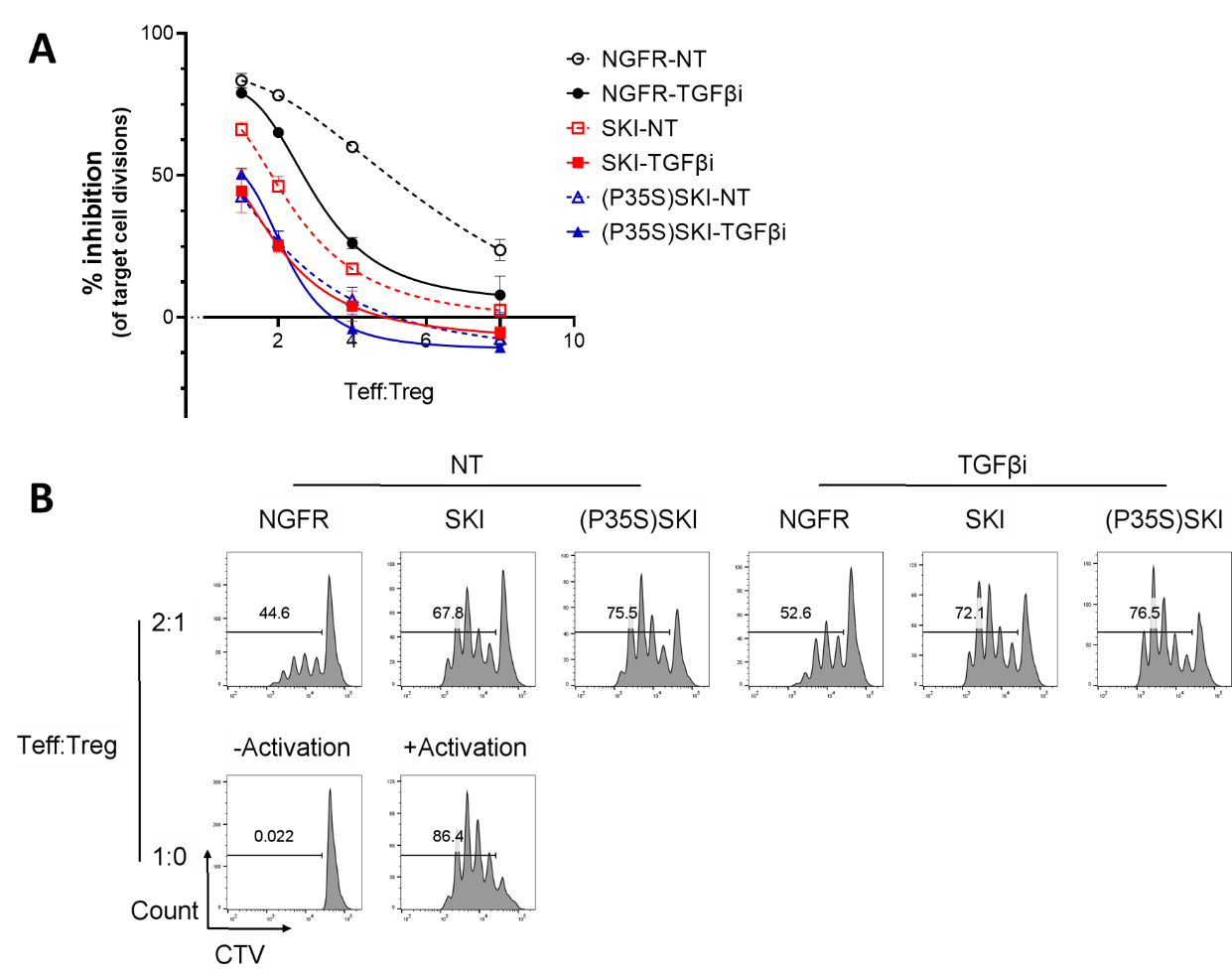


**Supplementary Figure 3. (P35S)SKI interrupts Treg suppression ability.** nTreg cells carrying T2A-NGFR empty vector (NGFR) or SKI-T2A-NGFR (SKI) or (P35S)SKI-T2A-NGFR ((P35S)SKI) were treated with (TGFβi) or without (NT) TGFβR1 inhibitor for three weeks, and then subjected to an *in vitro* suppression assay. Treg cells were cultured with Teff cells at the indicated ratios (Treg:Teff) for four days. Following incubation, Teff proliferation was assessed by CTV dilution and flow cytometry. **(A)** Suppression efficiency was calculated as “% inhibition of Teff division,” determined by the percentage reduction in the division index of Teff cells compared to Teff cells cultured alone. Data are presented as Mean ± SD. ^∗^p < 0.05, two-sided t test. n = 3 technical replicates of one donor. This experiment was independently reproduced with cells from two donors. **(B)** A representative CTV profile of Teff cells. This setup is representative of experiments conducted in two donors.


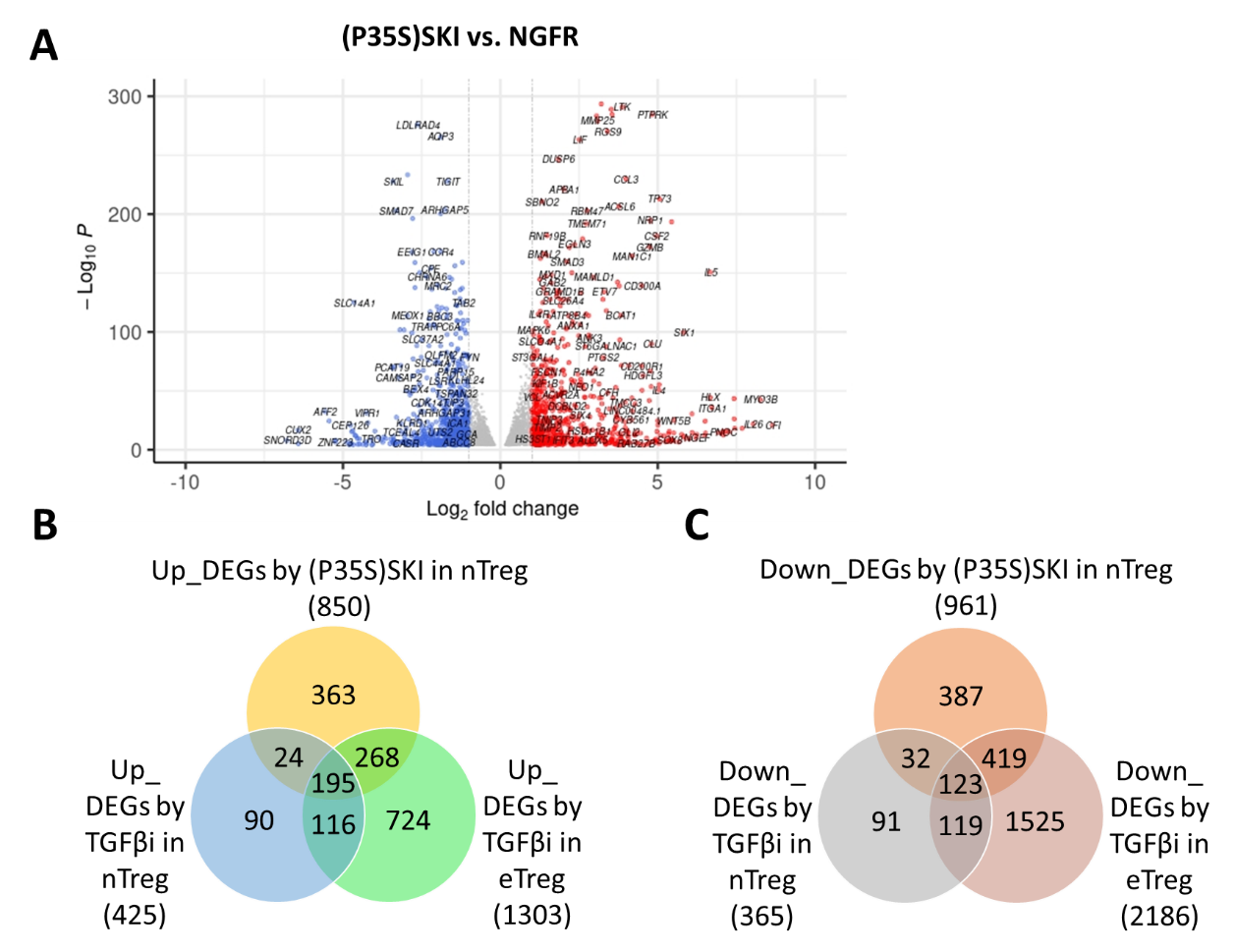


**Supplementary Figure 4. Identification of commonly up- and down-regulated genes by (P35S)SKI overexpression and TGFβi in Treg cells. (A)** Volcano plot of differently expressed genes (DEGs), adjusted p<0.05, |FC|>2, between (P35S)SKI and NGFR transduced nTreg cells. Red dots are up-regulated genes and blue dots are down-regulated genes by (P35S)SKI. **(B)** Overlap analysis generating 195 commonly up-regulated genes by SKI overexpression and TGFβi in Treg cells (Up_SKI/TGFβi) (Upregulated DEGs by (P35S)SKI in nTregs ∧ Upregulated DEGs by TGFβi in nTregs ∧ Upregulated DEGs by TGFβi in eTregs, adjusted p < 0.05, |FC| > 2). **(C)** Overlap analysis generating 123 commonly down-regulated genes by SKI overexpression and TGFβi in Treg cells (Down_SKI/TGFβi) (Downregulated DEGs by (P35S)SKI in nTregs ∧ Downregulated DEGs by TGFβi in nTregs ∧ Downregulated DEGs by TGFβi in eTregs, adjusted p < 0.05, |FC| > 2).


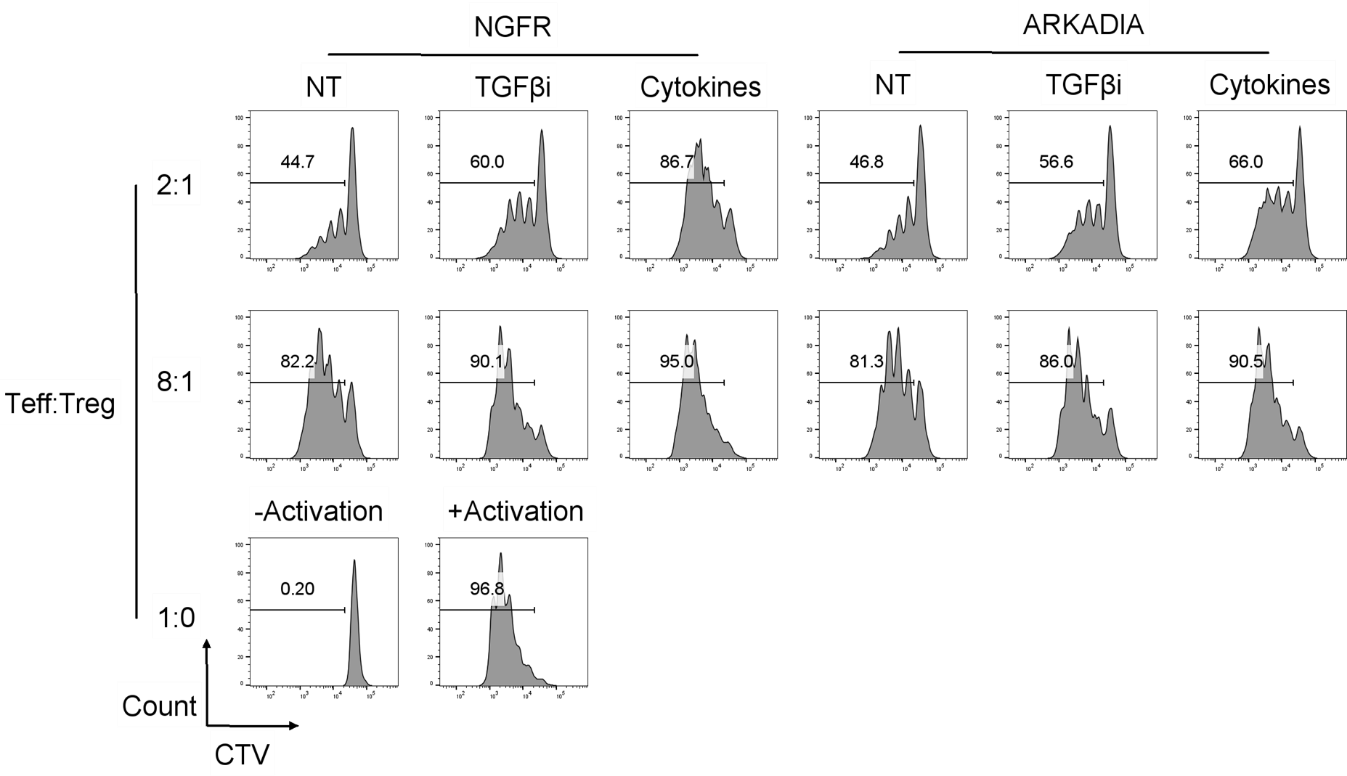


**Supplementary Figure 5.** nTreg cells carrying T2A-NGFR empty vector (NGFR) or ARKADIA-T2A-NGFR (ARKADIA) were left untreated (NT) or treated with inflammatory cytokines (Cytokines) or with TGFβR1 inhibitor (TGFβi) for three weeks. After the treatment, those nTreg cells were subjected to an *in vitro* suppression assay, by being cultured with Teff cells at a 2:1 ratio (Treg:Teff) or 8:1 ratio (Treg:Teff) for four days. Following incubation, Teff proliferation was assessed by CTV dilution and flow cytometry. A representative CTV profile of Teff cells. This setup is representative of experiments conducted in three donors.
