# Supplementary figures and images for: Differential regulation of Treg stability in human naïve and effector Treg subsets by TGFβ-signaling via ARKADIA-SKI axis
Source: Front Immunol. 2025 Sep 9;16:1636434. doi: 10.3389/fimmu.2025.1636434 (PMC12454061; doi:10.3389/fimmu.2025.1636434)

Figure 2A

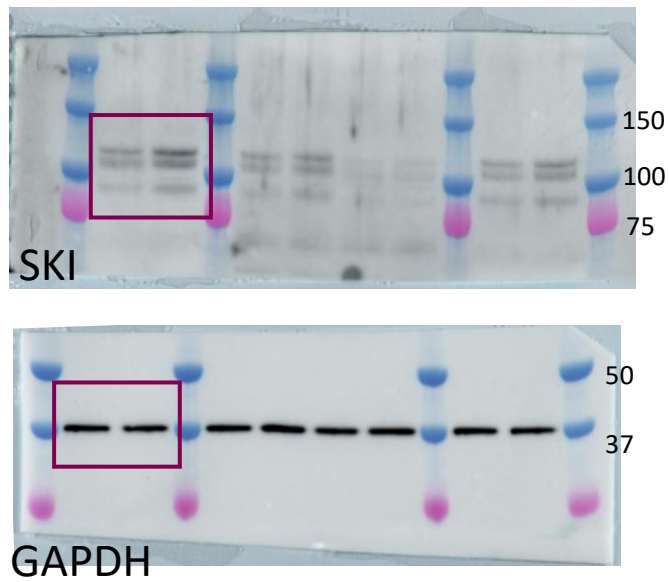

Figure 2C left

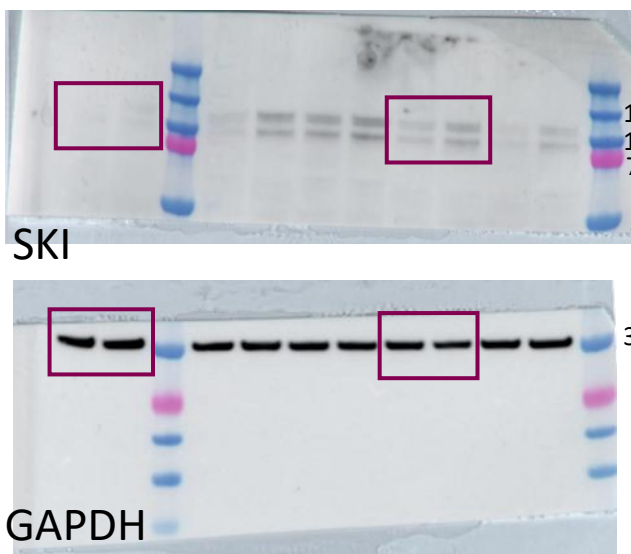

Figure 2C right

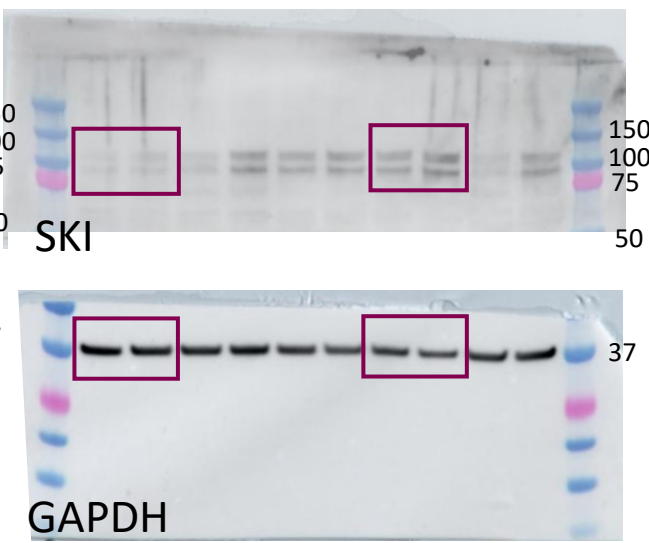

Supplementary Figure 2C

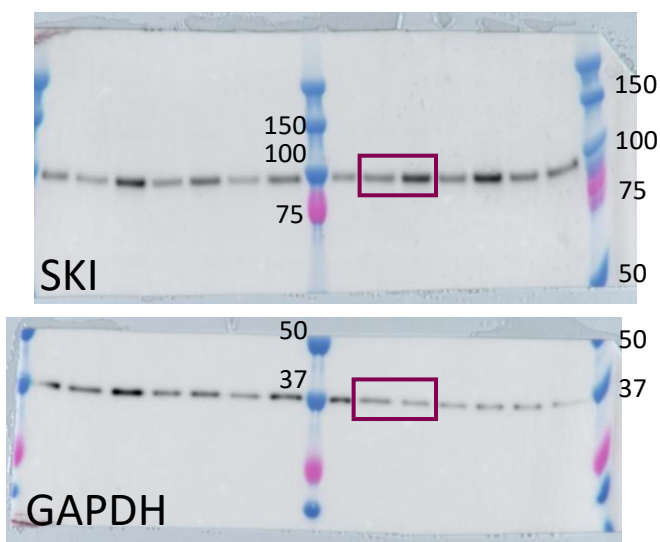

Figure 3A

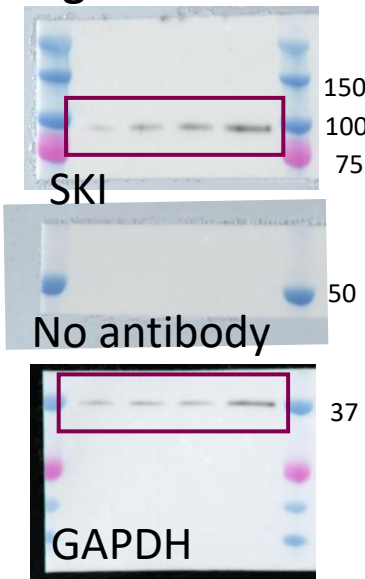

Figure 5A

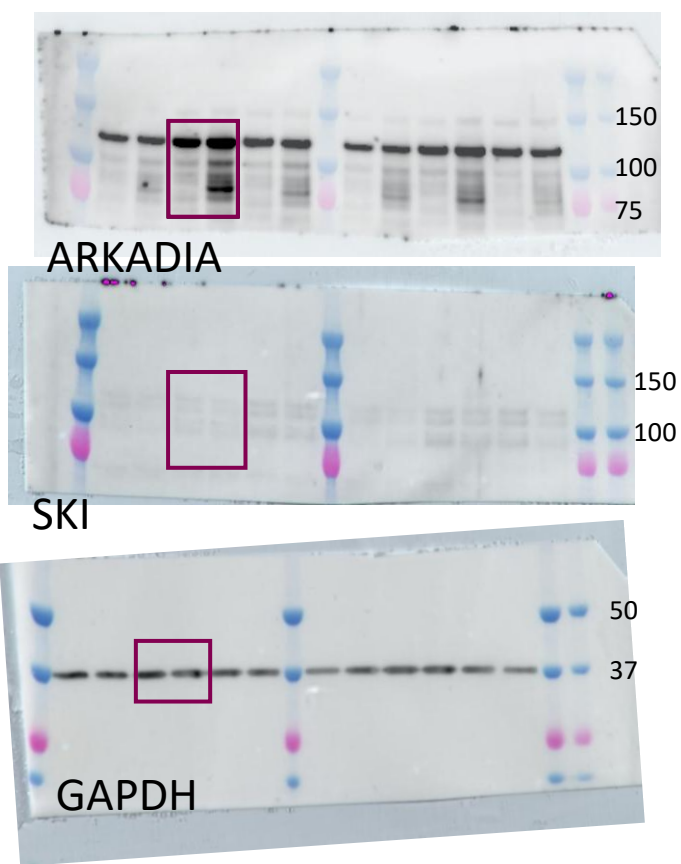

Supplement: Supplementary file 4 [file DataSheet2.pdf]
